# Non-contact detection of post-regurgitation deep inhalation in calves using infrared thermography and deep learning-based nostril segmentation

**DOI:** 10.1186/s12917-026-05340-y

**Published:** 2026-02-06

**Authors:** Sueun Kim, Norio Yamagishi, Shingo Ishikawa, Shinobu Tsuchiaka

**Affiliations:** Laboratory of Large Animal Clinical Medicine, Graduate School of Veterinary Sciences, Osaka Metropolitan University, Osaka, Japan

**Keywords:** Infrared thermography, Deep learning, Nostril segmentation, Breathing pattern, Rumination, Post-regurgitation deep inhalation, Non-contact monitoring

## Abstract

Continuous monitoring of rumination is highly informative for assessing cattle health and welfare. Traditional methods for rumination detection, such as pressure sensors, accelerometers, and acoustic sensors, require direct attachment to animals, which can be costly and stressful for the animals. This study proposes a non-contact approach for characterizing post-regurgitation deep inhalation (PRDI) in calves using infrared thermography and deep learning-based nostril segmentation. Synchronized RGB and temperature data were collected from 8 calves across 28 imaging sessions, during which rumination was visually confirmed. Deep learning algorithms were used to automatically segment the nostril region in each RGB frame, enabling temperature data extraction from the segmented regions to obtain breathing patterns. Visual observation of the video recordings was used to annotate the timing of regurgitation within the breathing patterns. Breathing patterns were analyzed to distinguish PRDI from other inhalation events not associated with rumination (non-rumination inhalation, NRI), with particular attention to deeper inspiratory minima that occur immediately after regurgitation. Statistical analysis demonstrated that PRDI events exhibit significantly deeper minima compared to NRI (*p* < 0.001). An optimal threshold for distinguishing PRDI from NRI within the breathing patterns was identified, achieving a balanced accuracy and G-mean of 0.72, with an area under the receiver operating characteristic curve (AUC) of 0.76. This study is preliminary in nature, largely due to the short recording durations and limited sample size, both of which inherently constrain the robustness and generalizability of the results. Nevertheless, the findings provide clear proof-of-concept evidence that post-regurgitation respiratory features can be detected using a fully non-contact approach.

## Introduction

Rumination is a process in ruminant animals such as cattle, in which previously ingested feed is brought back up into the mouth (regurgitation), chewed again (remastication), mixed with saliva (resalivation), and swallowed once more (redeglutition). This process helps reduce the particle size of ingested feed and increases saliva secretion, which buffers the rumen environment and optimizes the digestion of fibrous material. Meanwhile, diseases reduce rumination through similar mechanisms involving pain, discomfort, or impaired gastrointestinal function. Ruminal acidosis and displaced abomasum decreases rumination via pain and disrupted motility [1]. Periparturient diseases such as ketosis and mastitis also lower rumination time because of metabolic stress and reduced appetite [2, 3]. Welfare-related stressors, particularly heat stress, leads to reduced feed intake, shorter lying time, and elevated cortisol levels, which together impair gastrointestinal motility and decrease rumination time [4–6]. Because rumination reflects both health and welfare status, considerable efforts have been made to develop automated systems that can measure rumination time.

Generally, there are two main approaches to the automated monitoring of rumination time. The first method is based on directly detecting the regular, rhythmic movements associated with rumination. This approach includes the use of pressure sensors attached to the nose [7–9], three-axis accelerometers mounted on the ear [10], neck collar [11], or halter [12], as well as camera-based monitoring systems [13]. These devices measure the time cattle spend remasticating by identifying characteristic movement patterns that occur during rumination. The second method focuses on detecting regurgitation rather than remastication. This approach is indirect and often utilizes acoustic sensors [14, 15], such as microphones attached to a neck collar, to capture the sounds associated with regurgitation during rumination. In addition, one study [16] inferred rumination by identifying the absence of vertical (Z-axis) head movement and temporary pauses in breathing, which are physiological characteristics occurring during regurgitation. However, it remains unclear whether the achieved detection accuracy was primarily driven by breathing patterns, head movement, or a combination of both.

Recent advances in artificial intelligence-based image segmentation technology—where specific regions of an image, such as a cow’s nostrils, are automatically identified and outlined at the pixel level—have made it possible to automatically identify and extract a cow’s nostrils from images. Moreover, the nostril area itself provides a physiologically informative thermal signal: during inspiration, cold ambient air enters the nasal cavity and lowers the surface temperature around the nostrils, whereas during expiration, warm exhaled air raises the surface temperature. These temperature fluctuations can be clearly captured by infrared imaging [17]. In fact, a previous study using deep-learning-based nostril segmentation combined with infrared temperature data successfully obtained breathing patterns and demonstrated that this signal is highly relevant for detecting breathing events [18]. Unlike conventional monitoring systems that require physical attachments, this technique requires less animal handling, hence improve animal welfare.

Notably, rumination is accompanied by distinct respiratory characteristics, including brief apnea during regurgitation and a compensatory deep inspiration immediately afterward. The rumination begins with the regurgitation of a food bolus from the rumen, which is achieved through a coordinated series of physiological events, including an extra contraction of the reticulum, relaxation of the cardia, and a deep inspiratory movement of the ribs with the glottis closed [19]. The closure of the glottis during deep inspiration is a crucial element of this mechanism, as it creates the negative pressure required for efficient regurgitation of the bolus. Importantly, because the glottis remains closed during this inspiratory effort, airflow does not occur, and a brief cessation of respiratory airflow accompanies the regurgitation phase [20]. Furthermore, during redeglutition following remastication, several muscles involved in swallowing also serve respiratory functions. The recruitment of these shared muscles can lead to transient interruptions of breathing, contributing to short periods of apnea during the rumination cycle [21]. Once remastication resumes and these muscles return to their respiratory function, cattle typically exhibit a compensatory deep inspiratory movement. In this study, we focus on the deep inspiratory movement that occurs immediately after regurgitation and refer to this event as post-regurgitation deep inhalation (PRDI), an operationally defined respiratory feature that is not explicitly distinguished in the existing literature. All other detected inhalation events not temporally associated with regurgitation were classified as non-rumination inhalation (NRI). Accordingly, the objective of this study was to determine whether this PRDI can be distinguished from NRI using thermal breathing signals derived from infrared imaging and deep-learning-based nostril segmentation. To address this objective, we (1) examined whether PRDI differs significantly from NRI in terms of inspiratory depth, and (2) identified an optimal threshold for discriminating the two breathing types and evaluated its classification performance within continuous breathing patterns.

## Materials and methods

### Animals and devices

This study was conducted using 11 calves (12–14 weeks old) housed at the Kobe University farm (Food Resources Education and Research Center, Graduate School of Agricultural Science, Kobe University). The calves were housed in a semi-open barn in which one side was fully open to the external environment. Each calf was imaged three times at four-week intervals from February to September (ambient temperature range: 9–34.1 °C), resulting in a total of 33 imaging samples. Imaging was performed between 11:00 and 13:00 during the post-feeding period when rumination typically occurs. For each session, 1–2 min of thermal imaging and simultaneous RGB video (1440 × 1080 pixels) were acquired using a mobile infrared camera system (FLIR One Pro for iOS; emissivity 0.95, accuracy ± 3.0 °C, sensitivity 0.07 °C, field of view 55° × 43°, thermal resolution 160 × 120 pixels, temperature range − 20–400 °C; FLIR Systems Inc., Santa Barbara, CA, USA). The infrared module was attached to a smartphone and held by the operator without the use of a tripod. To minimize disturbance, the operator approached the calves slowly and conducted imaging at a distance of approximately 1.0–1.5 m. Recordings were obtained by manually orienting the camera toward the calf from a frontal or slightly oblique angle to ensure that the nostril region remained within the field of view. Rumination activity was visually identified in 28 of the 33 imaging sessions, and these samples were used for subsequent breathing pattern analysis. The raw imaging data used in this study originate from the same recording sessions as in our previous publication [22]. However, the analytical objective and labeling strategy are entirely different. The previous studies analyzed temperature changes in the eye and muzzle regions using their respective region of interests (ROIs), whereas the present study employs a nostril-specific ROI to extract and analyze breathing patterns.

### Data processing

#### Nostril temperature time-series extraction

**Fig. 1:**
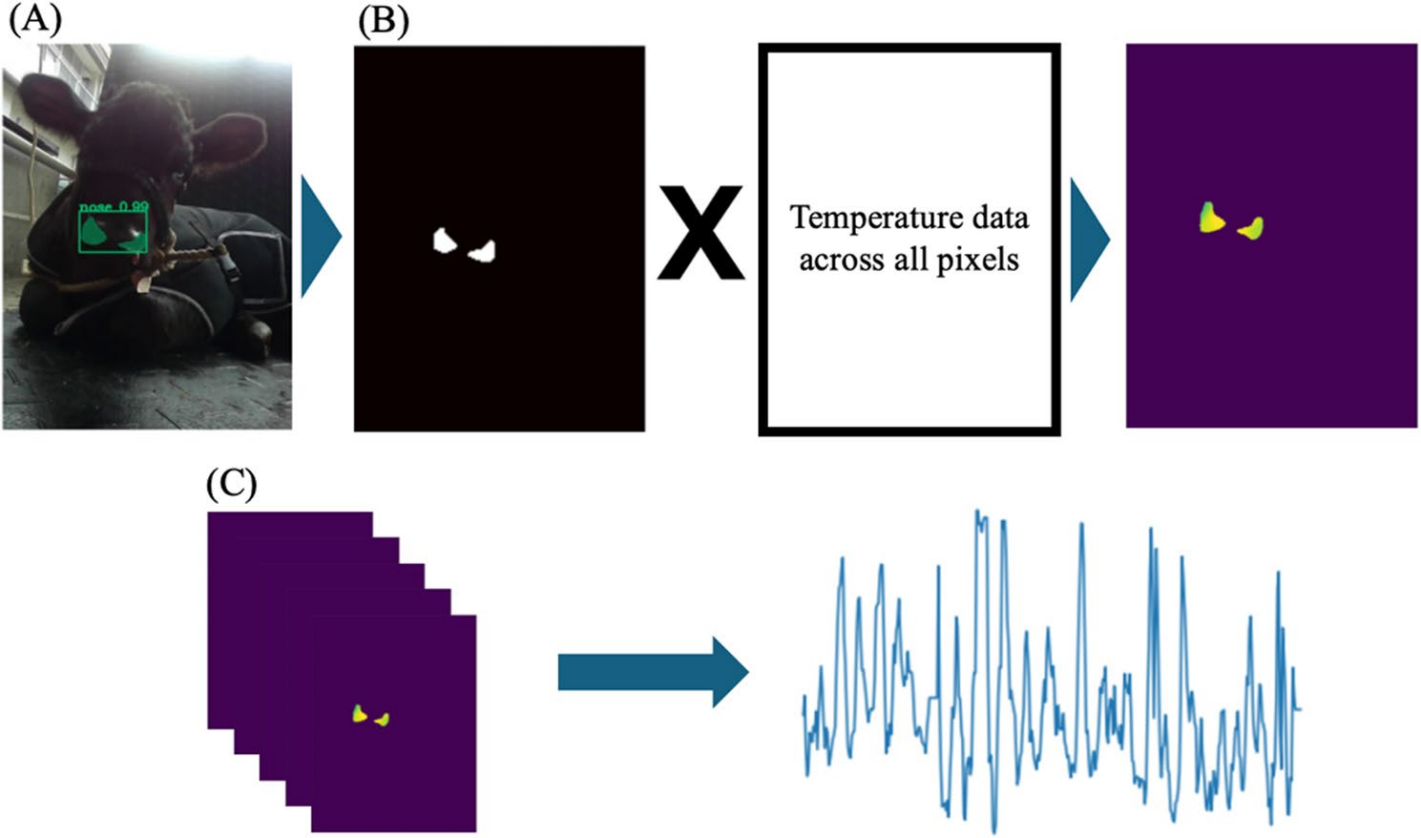
Workflow for ROI (nostril) extraction and nostril temperature time-series generation. **A** RGB image of a calf with automatically detected nostril regions, highlighted by green hatch patterns indicating segmentation results from the Mask R-CNN model. **B** The segmentation process showing the binary mask of detected nostril regions (left) combined with temperature data across all pixels (right) to extract temperature values specifically from the nostril areas (far right). **C** Sequential processing of multiple frames to generate continuous nostril temperature time-series data (x-axis: time, y-axis: temperature), which serve as the basis for subsequent breathing pattern analysis

The procedures for ROI extraction (nostril) and temperature data derivation were conducted as follows. First, nostril regions within each RGB frame were automatically detected (Fig. 1A) using a deep-learning segmentation model [18]. For model development, a total of 1,400 RGB images were manually annotated at the pixel level, of which 1,000 images were used for training and 400 images for validation. Annotation was performed by a single trained researcher using the VGG Image Annotator (VIA) with pixel-level polygon annotation. The nostril region was defined as the visible external nasal openings, excluding surrounding hair or muzzle surface, and both left and right nostrils were included when present. Frames with severe motion blur or complete nostril occlusion were excluded from annotation. The training and validation datasets were split at the frame level, while ensuring that frames from the same animal were not shared between two datasets. The segmentation model was implemented using Mask R-CNN [23] with a ResNet-101 backbone and COCO-based transfer learning, achieving a mean average precision (mAP) of 0.76 at an intersection-over-union (IoU) threshold 0.5 on the validation dataset. The model was trained with early stopping based on validation performance to prevent overfitting, and the reported mAP reflects the performance of a single-class segmentation task for the nostril region. Inference was performed on an AWS EC2 p2.xlarge instance equipped with a single NVIDIA Tesla K80 GPU (12 GB VRAM), an Intel Xeon E5-2686 v4 CPU (4 vCPUs), and 61 GiB of system memory, yielding an average inference time of 325 ms per image. Following segmentation, temperature values from the infrared camera were mapped onto the detected nostril mask, allowing extraction of temperature data corresponding precisely to the segmented nostril regions (Fig. 1B). This procedure was applied to all frames in each recording to generate continuous nostril temperature time-series data (Fig. 1C), which served as the raw physiological signals for subsequent breathing pattern analysis. This procedure was implemented in Python.

#### Breathing pattern processing

**Fig. 2:**
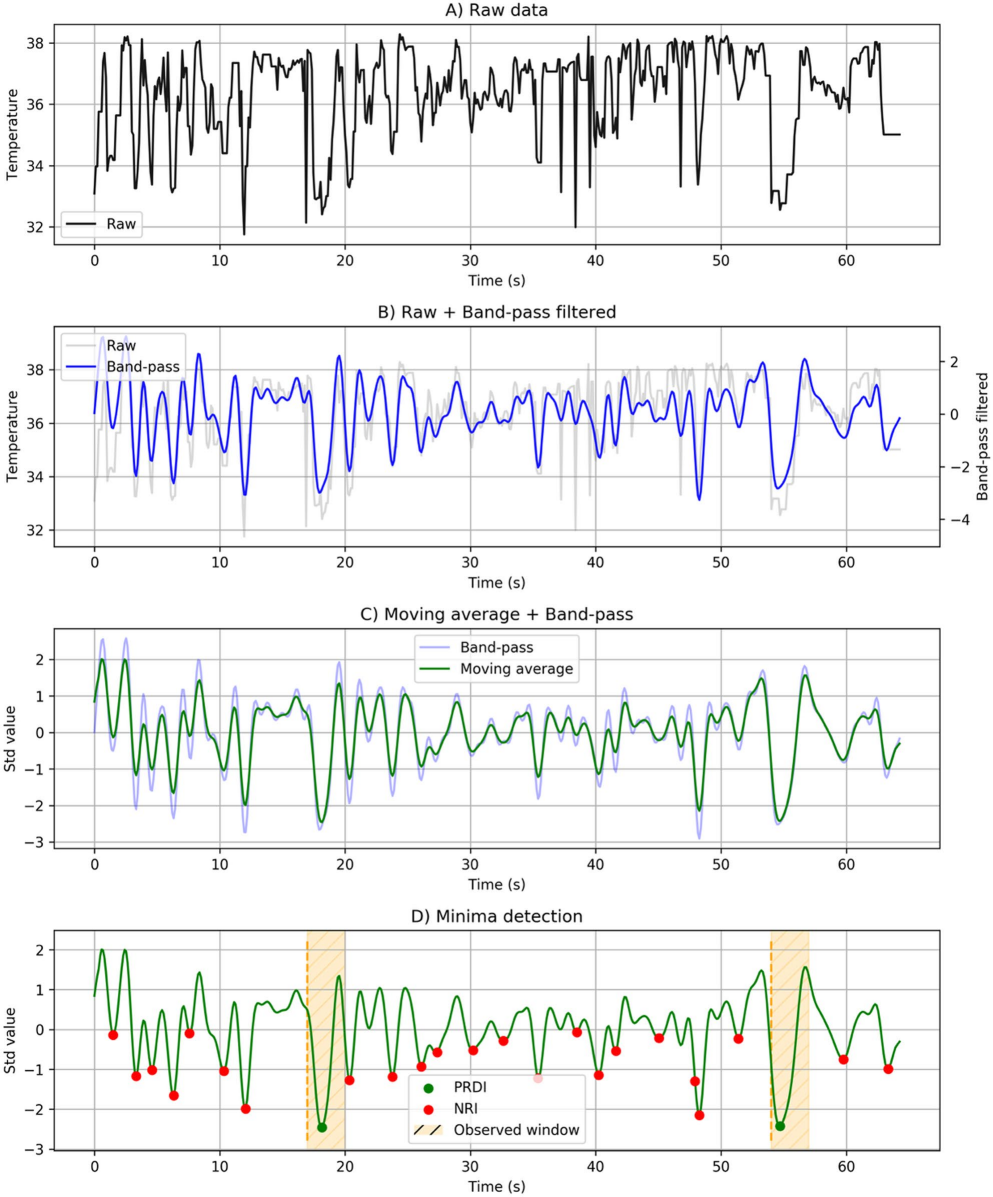
Detection of PRDI Events from Standardized Thermal Breathing Signals. This figure illustrates the signal processing steps and event classification used to characterize post-regurgitation deep inhalation (PRDI). **A** The raw nostril temperature time series extracted from the segmented nostril region. **B** Raw signal (gray) overlaid with the band-pass filtered signal (blue), applied to retain physiologically relevant breathing frequencies while suppressing low- and high-frequency noise. **C** Band-pass filtered signal (light blue) after z-score standardization and subsequent smoothing using a centered moving-average filter (green), resulting in a stabilized breathing signal. **D** Detection of local minima from the smoothed signal, corresponding to the transition from inspiration to expiration. Yellow shaded regions indicate the 3-s window immediately following each observed regurgitation event. Minima occurring within this window were classified as post-regurgitation deep inhalation (PRDI; green dots), whereas minima occurring outside the window were classified as non-rumination inhalation (NRI; red dots)

The nostril temperature time-series data obtained through the above procedures underwent several preprocessing steps to derive breathing patterns and identify characteristic rumination-related respiratory features. First, a bandpass filter was applied to the raw nostril temperature signals (Fig. 2A) to retain physiologically relevant breathing frequencies while suppressing high-frequency noise and low-frequency artifacts unrelated to respiration (Fig. 2B) [24]. In calves, the typical respiratory rate ranges approximately from 0.2 to 1.0 Hz (12–60 breaths per minute), and the filter cutoff values were selected to encompass this physiological range while attenuating components outside it. To mitigate scale variations across samples—arising from differences in absolute nostril temperature values due to ambient temperature, individual variability, and slight camera-animal distance differences—the band-pass filtered signals were standardized using z-score standardization. Subsequently, a centered moving average filter with a window size of 9 was applied (Fig. 2C). Unlike real-time running average filter that rely solely on past values, centered smoothing uses both past and future data points within the window. Because this study does not aim for real-time processing but instead conducts offline analysis, centered averaging was selected. Given that the sampling rate in this study was 8.7 data points per second, each window of 9 data points covers approximately 1.03 s of breathing data. This filtering approach calculates the mean of the data within a sliding window of approximately one second. Finally, breath events were identified by detecting local minima in the smoothed breathing patterns (Fig. 2D). These minima correspond to the transition points between inhalation and the onset of exhalation and were extracted for all samples to construct subsequent analysis. All data processing was performed in Python, using the following software environment and packages: Python 3.7.3, Numpy 1.16.4 [25], OpenCV 3.4.2 [26], scikit-learn [27], Matplotlib [28], and SciPy 1.3.1 [29].

#### Visual observations

To identify PRDI within the detected breathing patterns, visual observations were conducted via video recordings. The suitability of human observations as a reference method for rumination detection has been demonstrated in previous studies. The reliability of visual assessment is supported by a high Pearson correlation coefficient (*r* = 0.99) between different observers [30] and an inter-observer agreement rate of 99.8% [15]. Recent research also commonly uses human observation as the reference standard for rumination studies [7–12, 31, 32]. Regurgitation was defined as the moment when the cow interrupted chewing and, following redeglutition, brought a bolus back into the mouth for rechewing [16]. For each observed regurgitation, the 3-second period after the event was defined as the PRDI window (Fig. 2D, yellow shading), and any breathing event occurring within this window was classified as PRDI (Fig. 2D, green dots), while those that occurred outside this window were classified as NRI (Fig. 2D, red dots).

### Statistical analysis

To identify differences between PRDI and NRI and to assess whether PRDI can be detected within the overall breathing pattern, we performed two main statistical analyses. First, comparison of minima values between PRDI and NRI. We compared the depth of breathing events classified as PRDI versus NRI across all samples. As the results of the normality test indicated that the data were not normally distributed, we conducted a Mann-Whitney U-test to determine whether there was a statistically significant difference between the two groups. Second, receiver operating characteristic (ROC) analysis was performed to evaluate the overall discriminative ability of minima depth for distinguishing PRDI from NRI across all threshold values. The area under the ROC curve (AUC) was calculated as a threshold-independent measure of classification performance. Subsequently, to determine the optimal threshold for distinguishing PRDI from NRI based on the observed depth differences, we evaluated performance metrics across all possible threshold values as follows. For each threshold value, breathing events were classified as follows: If a breathing event predicted as PRDI (based on the threshold) fell within the predefined correct interval, it was counted as a true positive (TP). If it fell outside this interval, it was considered a false positive (FP). Conversely, if a candidate NRI event (e.g., a detected minimum that did not exceed the threshold) fell within the correct interval, it was counted as a false negative (FN); if it fell outside, it was a true negative (TN). Based on these classifications, we calculated standard performance metrics, including sensitivity and specificity.

$$\mathrm{Sensitivity}=\frac{TP}{TP+FN}$$

$$\mathrm{Specificity}=\frac{TN}{TN+FP}$$

Using these values, we computed the G-mean and balanced accuracy for each threshold. The G-mean is defined as the geometric mean of sensitivity and specificity, while balanced accuracy is the average of these two metrics.

$$\mathrm{G-mean}=\sqrt{sensitivity\times\:specificity}$$

$$\text{Balanced accuracy}=\frac{sensitivity+specificity}{2}$$

Subsequently, to determine the optimal threshold, we analyzed the curves of G-mean and balanced accuracy curves across the full range of candidate threshold values. The optimal threshold was selected as the value that maximized both G-mean and balanced accuracy simultaneously. When multiple adjacent threshold values yielded comparable maxima, the midpoint of this range was chosen to ensure robustness against minor signal fluctuations.

## Results

### Comparison of minima values between PRDI and NRI

**Fig. 3:**
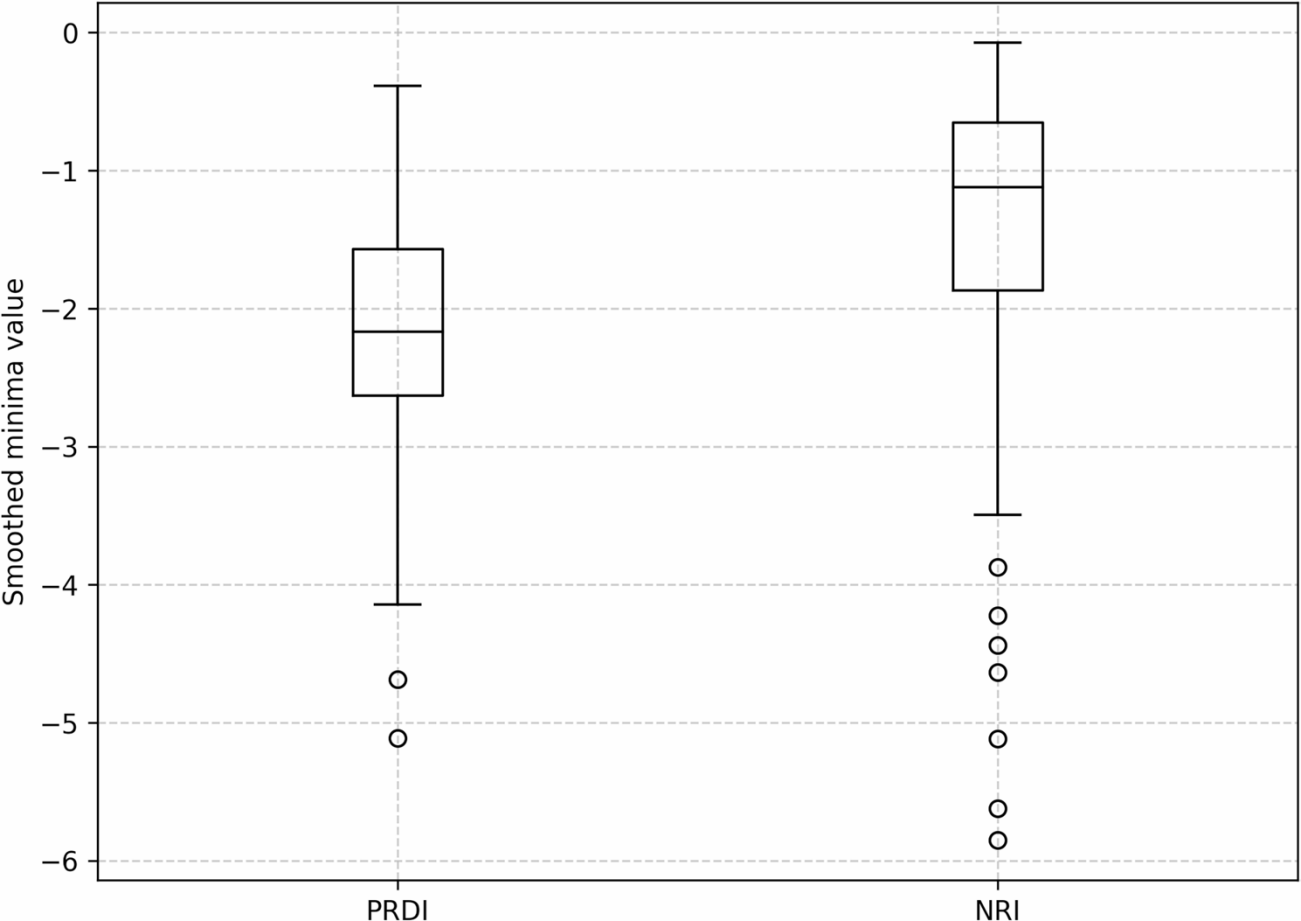
Smoothed minima values during PRDI and NRI. This boxplot compares the smoothed minima values of breathing events classified as post-regurgitation deep inhalation (PRDI) and non-rumination inhalation (NRI). PRDI events show significantly lower (more negative) minima values than NRI events (*p* < 0.001)

To evaluate the differences between PRDI and NRI events, we compared the smoothed minima values of breathing events classified as either within the regurgitation window (PRDI) or outside it (NRI). The boxplot (Fig. 3) demonstrates that PRDI events exhibit significantly lower (more negative) minima values compared to NRI events. Specifically, the median value for PRDI was approximately − 2.17, with an interquartile range extending from approximately − 2.63 to -1.57, while NRI showed a median of approximately − 1.12 with an interquartile range from − 1.87 to -0.66. This difference was statistically significant according to the Mann-Whitney U test (*p* < 0.001), indicating that minima depth differed between PRDI and NRI events.

### Determination of optimal threshold for PRDI detection

**Fig. 4:**
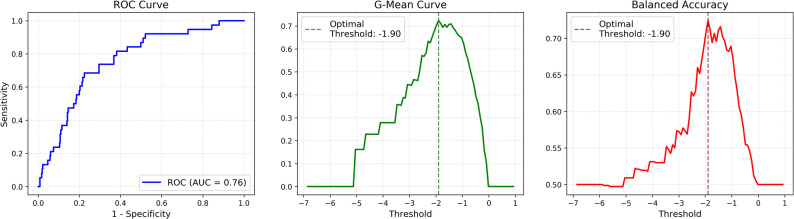
Threshold optimization and classification performance for PRDI detection. The receiver operating characteristic (ROC) curve (left) illustrates the overall discriminative performance for distinguishing post-regurgitation deep inhalation (PRDI) from non-rumination inhalation (NRI) across all threshold values, with an area under the curve (AUC) of 0.76. The G-Mean curve (middle) and balanced accuracy curve (right) show the performance of individual threshold values. Both curves reached their maximum at an optimal threshold of -1.90, achieving a G-Mean and balanced accuracy of 0.72

To establish an objective threshold for distinguishing PRDI from NRI based on the depth of the minima, ROC analysis was first performed to evaluate the overall discriminative ability across all possible threshold values. The ROC curve yielded an AUC of 0.76 (Fig. 4, left), indicating moderate discriminative performance of minima depth for separating PRDI from NRI. Subsequently, threshold-dependent performance metrics were evaluated to determine an optimal operating point. The G-Mean curve (Fig. 4, middle) and the balanced accuracy curve (Fig. 4, right) both show a clear peak at a threshold of approximately − 1.90. At this optimal threshold, the sensitivity and specificity for distinguishing PRDI events were 0.68 and 0.76, respectively, yielding a G-Mean and balanced accuracy of 0.72. These results indicate that the chosen threshold provides a balanced trade-off between correctly identifying PRDI events and minimizing false positives.

## Discussion

### Summary of findings and practical intent

The present study investigated whether PRDI—defined here as the deep inspiratory movement occurring immediately after regurgitation—can be distinguished from NRI using thermal breathing patterns extracted through infrared imaging and deep-learning-based nostril segmentation. Across all samples, PRDI events consistently exhibited deeper inspiratory minima than NRI, demonstrating that the physiological characteristics of rumination are sufficiently reflected in the nostril temperature signal. ROC analysis further demonstrated a moderate overall discriminative ability of minima depth (AUC = 0.76), supporting the feasibility of distinguishing PRDI-related respiratory features from other inhalation events using thermal breathing signals. By identifying an optimal threshold that maximized both sensitivity and specificity, we established a baseline method for distinguishing PRDI from NRI under a non-contact monitoring framework. A key motivation for developing this approach lies in its practical value. Conventional rumination detection typically depends on wearable sensors—such as noseband pressure sensors [7–9] or accelerometers [10–12]—mounted on individual animals. These devices, although highly accurate, require physical attachment, maintenance, and per-animal deployment, which may impose costs, labor, and stress. In contrast, imaging-based systems provide a fully non-contact alternative. Furthermore, by simply redefining the ROI—or by detecting multiple ROIs simultaneously within the same image—infrared imaging enables simultaneous measurement of additional biometric indicators, such as temperature changes around the eye and muzzle regions [22, 33], all of which are relevant to physiological and welfare assessments. Thus, the method presented here not only identifies rumination-related respiratory features but also contributes to a broader framework for multi-parameter monitoring in cattle.

### Technical rationale for the imaging and segmentation pipeline

Given that the primary objective of this study was to determine whether PRDI can be distinguished from NRI based on respiratory patterns, the imaging pipeline was intentionally designed to prioritize signal accuracy over real-time feasibility. To achieve this, we employed a combined infrared-RGB approach together with Mask R-CNN, which enables pixel-level segmentation of the nostril region. Although infrared imaging alone is theoretically sufficient for measuring nostril temperature changes, its low-texture nature often results in unstable nostril localization, particularly under varying head postures, partial occlusion, or motion blur [34]. These limitations can degrade the signal-to-noise ratio of the derived breathing pattern and compromise the accuracy of PRDI detection. To overcome these challenges, we adopted a combined infrared–RGB approach in which nostril segmentation was performed on RGB frames and subsequently mapped onto the corresponding thermal data. This strategy leverages the rich visual features available in RGB images while preserving the physiological value of the thermal signal, enhancing robustness to extreme head angles and partial occlusion—conditions that frequently impair IR-only approaches. In addition, Mask R-CNN was chosen as the segmentation model because it provides pixel-level masks rather than the bounding-box outputs generated by lighter detectors. Pixel-level segmentation ensures that temperature extraction is limited precisely to nostril pixels, minimizing contamination from surrounding facial regions with different thermal properties. This fine-grained localization improves the fidelity of the breathing waveform and reduces artifacts derived from bounding-box misalignment. However, for real-time use in farm settings, the pipeline should be simplified—for example by relying solely on infrared images and lighter detection models—which we consider an important direction for future work.

### Detection performance and metric selection

Beyond the design-related considerations, another important aspect of this study involves evaluating the classification performance for distinguishing PRDI, especially under conditions of physiological or behavioral variability. When selecting metrics to detect specific behaviors or conditions, commonly used indicators include sensitivity, positive predictive value, and F1 score. Previous studies have also focused on these metrics—sensitivity [35] and F1 score [14, 36]—for accurately detecting the presence of rumination. However, rumination is often reduced or absent during disease or stress [1–6], so effectively detecting its absence is particularly important. In such cases—as in this study—greater emphasis should be placed on specificity and negative predictive value. However, we encountered a significant data imbalance, with NRI far outnumbering PRDI in breathing patterns. Such imbalance can lead to underestimation of sensitivity and overestimation of specificity. To address this, we applied statistical analysis methods—including G-mean and balanced accuracy—that jointly consider both sensitivity and specificity [37]. When optimizing the threshold to achieve the best balance between sensitivity and specificity, a lower threshold increases the likelihood that both PRDI and NRI will be classified as PRDI. This results in a higher number of false positives, which decreases specificity but increases sensitivity. In other words, while this approach captures more true PRDI, it also misclassifies more NRI as PRDI. Conversely, a higher threshold increases the likelihood that some PRDI will be misclassified as NRI, raising the number of false negatives, decreasing sensitivity, and increasing specificity. Here, although fewer NRI events are misclassified, more PRDIs are missed. Given this inverse relationship between sensitivity and specificity with threshold adjustment, we used balanced statistical metrics—including G-mean and balanced accuracy—to reliably detect signals of rumination-related breathing signals, even in the presence of data imbalance.

### Limitations and directions for future research

There are several practical considerations related to the experimental design of this study, including the age of the animals, the relatively short observation period, the limited sample size, and the presence of individuals in which rumination behavior was not observed. First, the animals used in this study were 12- to 14-week-old post-weaning calves, approximately 2 to 6 weeks after weaning. It is important to note that calves at this developmental stage may not fully represent the rumination behavior and physiological characteristics of mature cattle. In this study, the rumination interval observed directly from the video recordings averaged approximately 37.5 ± 9.73 s, which is shorter than the 59.27 ± 9.01 s interval commonly reported in adult cattle [16]. This discrepancy is likely attributable to the smaller rumen capacity and lower digestive efficiency of calves, which results in the ingestion of smaller quantities per feeding and a tendency toward more frequent rumination cycles [38]. Although these characteristics may limit the generalizability of our findings, they do not represent a significant constraint in the context of this study, which aimed not to characterize rumination physiology per se, but rather to evaluate the feasibility of distinguishing PRDI based on breathing patterns. Nonetheless, it is possible that the respiratory features associated with regurgitation are more pronounced in adult cattle, and future studies involving larger sample sizes, animals of various age groups, and management conditions will be necessary to further assess the broader applicability and robustness of the detection method. In addition, in 3 out of the 11 calves, rumination behavior was not observed during the 1–2 min recording sessions. This absence is unlikely to be due to health issues or insufficient observation time, given that the observation-based rumination interval in this study averaged 37.5 ± 9.73 s. Instead, the lack of rumination may partly reflect normal behavioral variability. Because multiple calves were housed together within the same barn, a hand-held imaging approach was required to ensure that each target calf was appropriately captured, and the close proximity of the operator may have contributed to mild alertness in some calves. However, this limitation arises from the data-collection procedure rather than from any constraint of the infrared technology itself. In practical farm applications, if an individual identification algorithm is incorporated to distinguish multiple animals within the same frame, the system could reply on fixed, remotely positioned cameras, eliminating the need for human presence near the animals and thereby achieving the non-contact, low-stress monitoring described in the Introduction.

In terms of accuracy, previous studies on rumination detection in cattle have primarily utilized signals derived from remastication movements—specifically, the rhythmic motion of the jaw or head. For example, the noseband pressure sensor detects regular pressure changes during remastication by means of a pressure sensor attached to a noseband, achieving high accuracy in distinguishing rumination from eating (typically above 0.90, with some studies reporting values above 0.96 [8, 9]). Similarly, sensors equipped with accelerometers on the cow’s head or neck can detect the rhythmic movements of the head and jaw associated with rumination. Although the accuracy varies depending on the placement of the accelerometer, most studies report accuracies above 0.8 [10–12]. In contrast, the present study focuses on distinguishing post-regurgitation respiratory features associated with rumination based on thermal breathing patterns. By employing an infrared imaging camera and a deep learning-based segmentation method for the nostril region, we acquired breathing patterns from cattle in a non-contact manner and optimized a threshold to distinguish PRDI from NRI. When the proposed classification threshold (threshold − 1.90) was applied, both G-mean and balanced accuracy reached 0.72, which is somewhat lower than the values reported for movement-based signal methods, but it aligns with the accuracy reported for acoustic approaches (0.65–0.75) that also target regurgitation events rather than remastication [14, 15]. Plus, one study [16] using a combination of respiration-based pressure sensors and accelerometers to detect regurgitation achieved high accuracy by fusing both signal types. However, the study did not clearly report the performance of each individual signal source, making it unclear whether satisfactory detection could be achieved using only one type of signal. Importantly, we attempted to maximize robustness by employing a combined infrared-RGB pipeline and using Mask R-CNN for pixel-level segmentation, along with multiple signal-processing steps—including bandpass filtering and moving average filter—to reduce noise within the breathing patterns. Considering these steps, the difference in accuracy may not simply reflect shortcoming of the algorithm; rather, it may stem from a more fundamental methodological distinction. Movement-based systems detect rumination by leveraging the long, rhythmic, and repetitive nature of remastication, which spans many cycles and therefore enables robust temporal pattern recognition. In contrast, the present method—and acoustic studies—attempts to infer rumination from single physiological events, such as the brief apnea and compensatory inspiration associated with regurgitation, or the discrete sounds produced during bolus movement. Inferring rumination from such event-level features may inherently pose greater variability and thus limit attainable accuracy. If future analyses reveal that the breathing-based signal itself has inherent limitations, then incorporating multimodal strategies will be essential—for instance, combining respiratory features with visual tracking of head posture. Notably, during regurgitation, the head typically remains relatively stationary in the vertical (Z-axis) direction—a feature that has been captured using accelerometers [16]. However, this characteristic could also be extracted through computer vision–based approaches, allowing for non-contact visual tracking of head posture instead of relying on wearable sensors [39]. Integrating such complementary sources may overcome the signal fragility of breathing data alone, moving the system closer toward practical, real-time applications on commercial farms.

## Conclusion

This study demonstrated that non-contact differentiation between PRDI and NRI in calves is feasible using infrared thermography and deep learning-based nostril segmentation, even within short two-minute recordings obtained while the animal was quietly lying down. In particular, post-regurgitation inhalation events were characterized by lower nostril minimum temperatures than other inhalation events, which were consistently reflected as deeper minima in the nostril temperature–derived breathing signal. Statistical analyses confirmed that PRDI events exhibit significantly deeper respiratory minima compared to NRI. The optimized classification threshold for detecting PRDI achieved a balanced accuracy and G-mean of 0.72, indicating moderate but meaningful discriminative capability for this newly defined respiratory feature. While these findings confirm the basic feasibility of distinguishing PRDI from NRI under stable conditions, additional work is required to determine how this approach could be implemented for continuous monitoring in real farm environments.

## Data Availability

The datasets generated and/or analyzed during the current study are available from the corresponding author on reasonable request.

## References

[1] Stangaferro ML, Wijma R, Caixeta LS, Al-Abri MA, Giordano JO. Use of rumination and activity monitoring for the identification of dairy cows with health disorders: part I. Metabolic and digestive disorders. J Dairy Sci. 2016;99(9):7395–410. 10.3168/jds.2016-10907. 27372591 10.3168/jds.2016-10907

[2] Soriani N, Trevisi E, Calamari L. Relationships between rumination time, metabolic conditions and health status in dairy cows during the transition period. J Anim Sci. 2012;90(12):4544–54. 10.2527/jas.2011-5064. 23255819 10.2527/jas.2012-5064

[3] Cocco R, Canozzi MEA, Fischer V. Rumination time as an early predictor of metritis and subclinical ketosis in dairy cows at the beginning of lactation: systematic review–meta-analysis. Prev Vet Med. 2021;189:105309. 10.1016/j.prevetmed.2021.105309. 33689960 10.1016/j.prevetmed.2021.105309

[4] Soriani N, Panella G, Calamari L. Rumination time during the summer season and its relationships with metabolic conditions and milk production. J Dairy Sci. 2013;96(8):5082–94. 10.3168/jds.2013-6620. 23791488 10.3168/jds.2013-6620

[5] Abeni F, Galli A. Monitoring cow activity and rumination time for an early detection of heat stress in dairy cows. Int J Biometeorol. 2017;61(3):417–25. 10.1007/s00484-016-1222-z. 27498881 10.1007/s00484-016-1222-z

[6] Moretti R, Biffani S, Chessa S, Bozzi R. Heat stress effects on Holstein dairy cows’ rumination. Animal. 2017;11(12):2320–5. 10.1017/S1751731117001173. 28578746 10.1017/S1751731117001173

[7] Braun U, Trösch L, Nydegger F, Hässig M. Evaluation of eating and rumination behaviour in cows using a noseband pressure sensor. BMC Vet Res. 2013;9:164. 10.1186/1746-6148-9-164. 23941142 10.1186/1746-6148-9-164 PMC3751437

[8] Zehner N, Umstätter C, Niederhauser JJ, Schick M. System specification and validation of a noseband pressure sensor for measurement of ruminating and eating behavior in stable-fed cows. Comput Electron Agric. 2017;136:31–41. 10.1016/j.compag.2017.02.021.

[9] Li Z, Cheng L, Cullen B. Validation and use of the RumiWatch noseband sensor for monitoring grazing behaviours of lactating dairy cows. Dairy. 2021;2(1):104–11. 10.3390/dairy2010010.

[10] Bikker JP, van Laar H, Rump P, Doorenbos J, van Meurs K, Griffioen GM, Dijkstra J. Evaluation of an ear-attached movement sensor to record cow feeding behavior and activity. J Dairy Sci. 2014;97(5):2974–9. 10.3168/jds.2013-7560. 24630647 10.3168/jds.2013-7560

[11] Leso L, Becciolini V, Rossi G, Camiciottoli S, Barbari M. Validation of a commercial collar-based sensor for monitoring eating and ruminating behaviour of dairy cows. Animals. 2021;11(10):2852. 10.3390/ani11102852. 34679872 10.3390/ani11102852 PMC8532760

[12] Ding L, Lv Y, Jiang R, Zhao W, Li Q, Yang B, et al. Predicting the feed intake of cattle based on jaw movement using a triaxial accelerometer. Agriculture. 2022;12(7):899. 10.3390/agriculture12070899.

[13] Li J, Liu Y, Zheng W, Chen X, Ma Y, Guo L. Monitoring cattle ruminating behavior based on an improved keypoint detection model. Animals. 2024;14(12):1791. 10.3390/ani14121791. 38929410 10.3390/ani14121791 PMC11200719

[14] Chelotti JO, Vanrell SR, Rau LSM, Galli JR, Planisich AM, Utsumi SA, et al. An online method for estimating grazing and rumination bouts using acoustic signals in grazing cattle. Comput Electron Agric. 2020;173:105443. 10.1016/j.compag.2020.105443.

[15] Elischer MF, Arceo ME, Karcher EL, Siegford JM. Validating the accuracy of activity and rumination monitor data from dairy cows housed in a pasture-based automatic milking system. J Dairy Sci. 2013;96(10):6412–22. 10.3168/jds.2013-6790. 23958013 10.3168/jds.2013-6790

[16] Hoffmann G, Strutzke S, Fiske D, Heinicke J, Mylostyvyi R. A new approach to recording rumination behavior in dairy cows. Sensors. 2024;24(17):5521. 10.3390/s24175521. 39275432 10.3390/s24175521 PMC11398270

[17] Lowe G, Sutherland M, Waas J, Schaefer A, Cox N, Stewart M. Infrared thermography—A non-invasive method of measuring respiration rate in calves. Animals. 2019;9(8):535. 10.3390/ani9080535. 31394802 10.3390/ani9080535 PMC6720651

[18] Kim S, Hidaka Y. Breathing pattern analysis in cattle using infrared thermography and computer vision. Animals. 2021;11(1):207. 10.3390/ani11010207. 33466995 10.3390/ani11010207 PMC7830257

[19] Klein BG. Cunningham’s textbook of veterinary physiology. 5th ed. St. Louis, MO: Elsevier Saunders; 2012. ISBN: 978-1-4377-2361-8.

[20] Reece WO, Rowe EW. Functional anatomy and physiology of domestic animals. 5th ed. Hoboken, NJ: John Wiley & Sons, Inc.; 2017. ISBN:978-1-119-27084-3.

[21] Shaker R, Belafsky PC, Postma GN, Easterling C, editors. Principles of deglutition: a multidisciplinary text for swallowing and its disorders. New York: Springer; 2013. 10.1007/978-1-4614-3794-9.

[22] Kim S, Yamagishi N, Ishikawa S, Tsuchiaka S. Unique temperature change patterns in calves’ eyes and muzzles: a non-invasive approach using infrared thermography and object detection. Front Vet Sci. 2025;12:1548906. 10.3389/fvets.2025.1548906. 40129574 10.3389/fvets.2025.1548906 PMC11931118

[23] He K, Gkioxari G, Dollár P, Girshick R, Mask R-CNN. In: Proceedings of the IEEE International Conference on Computer Vision (ICCV). 2017. pp. 2961–2969. 10.1109/ICCV.2017.322.

[24] Wang J, Liu L, Lu M, Okinda C, Lovarelli D, Guarino M, et al. The Estimation of broiler respiration rate based on the semantic segmentation and video amplification. Front Physiol. 2022;10:1047077. 10.3389/fphy.2022.1047077.

[25] Harris CR, Millman KJ, van der Walt SJ, et al. Array programming with numpy. Nature. 2020;585:357–62. 10.1038/s41586-020-2649-2. 32939066 10.1038/s41586-020-2649-2 PMC7759461

[26] OpenCV. Open Source Computer Vision Library. https://opencv.org/.

[27] Pedregosa F, Varoquaux G, Gramfort A, et al. Scikit-learn: machine learning in python. J Mach Learn Res. 2011;12:2825–30.

[28] Hunter JD, Matplotlib. A 2D graphics environment. Comput Sci Eng. 2007;9(3):90–5. 10.1109/MCSE.2007.55.

[29] Virtanen P, Gommers R, Oliphant TE, et al. SciPy 1.0: fundamental algorithms for scientific computing in python. Nat Methods. 2020;17:261–72. 10.1038/s41592-019-0686-2. 32015543 10.1038/s41592-019-0686-2 PMC7056644

[30] Schirmann K, von Keyserlingk MA, Weary DM, Veira DM, Heuwieser W. Validation of a system for monitoring rumination in dairy cows. J Dairy Sci. 2009;92(12):6052–5. 10.3168/jds.2009-2361. 19923608 10.3168/jds.2009-2361

[31] Eslamizad M, Tümmler LM, Derno M, Hoch M, Kuhla B. Development of a pressure sensor-based system for measuring rumination time in pre-weaned dairy calves. J Anim Sci. 2018;96(11):4483–9. 10.1093/jas/sky337. 30256955 10.1093/jas/sky337 PMC6247832

[32] Wang Y, Chen T, Li B, Li Q. Automatic identification and analysis of multi-object cattle rumination based on computer vision. J Anim Sci Technol. 2023;65(3):519–34. 10.5187/jast.2022.e87. 37332285 10.5187/jast.2022.e87 PMC10271932

[33] Kim S, Yamagishi N, Ishikawa S, Tsuchiaka S. AI-enhanced infrared thermography for reliable detection and Spatial mapping of temperature patterns in calf eyes and muzzles. BMC Vet Res. 2025;21(1):468. 10.1186/s12917-025-04919-1. 40665350 10.1186/s12917-025-04919-1 PMC12261661

[34] Eom JS, Park MS, Yang HR, Eum YJ, Sohn JH. Nose detection and breathing monitoring in thermal images. Int J Adv Sci Technol. 2017;109:67–76. 10.14257/ijast.2017.109.07.

[35] Song X, Van der Tol PPJ, Koerkamp PG, Bokkers EAM. Hot topic: automated assessment of reticulo-ruminal motility in dairy cows using 3-dimensional vision. J Dairy Sci. 2019;102(10):9076–81. 10.3168/jds.2019-16550. 31400896 10.3168/jds.2019-16550

[36] Hamilton AW, Davison C, Tachtatzis C, Andonovic I, Michie C, Ferguson HJ, et al. Identification of the rumination in cattle using support vector machines with motion-sensitive bolus sensors. Sensors. 2019;19(5):1165. 10.3390/s19051165. 30866541 10.3390/s19051165 PMC6427569

[37] Akosa J. Predictive accuracy: a misleading performance measure for highly imbalanced data. In: Proceedings of the SAS Global Forum. Cary, NC: SAS Institute Inc.; 2017. Paper 942–2017. https://support.sas.com/resources/papers/proceedings17/0942-2017.pdf

[38] Schwarzkopf S, Kinoshita A, Hüther L, Salm L, Kehraus S, Südekum KH, et al. Weaning age influences indicators of rumen function and development in female Holstein calves. BMC Vet Res. 2022;18:102. 10.1186/s12917-022-03163-1. 35300681 10.1186/s12917-022-03163-1 PMC8928593

[39] Kim S. Automated cattle head and ear pose Estimation using deep learning for animal welfare research. Vet Sci. 2025;12(7):664. 10.3390/vetsci12070664. 40711324 10.3390/vetsci12070664 PMC12300177

